# Diagnostic challenges and forensic implications in a case of infantile fatal myocarditis

**DOI:** 10.1007/s12024-023-00659-6

**Published:** 2023-06-19

**Authors:** Federica Grimaldi, Maria Paola Bonasoni, Guido Pelletti, Liliana Gabrielli, Susi Pelotti

**Affiliations:** 1 Unit of Legal Medicine, Department of Medical and Surgical Science, University of Bologna, Bologna, 40126 Italy; 2 Pathology Unit, Azienda Unità Sanitaria Locale - IRCCS di Reggio Emilia, Reggio Emilia, 42122 Italy; 3 Microbiology Unit, IRCCS Azienda Ospedaliero-Universitaria di Bologna, Bologna, 40138 Italy

**Keywords:** Myocarditis, Child, Sudden death, Autopsy, Forensic medicine, RT-PCR, Histology

## Abstract

We present the case of a 23-month-old child who died less than 24 h after the onset of cardiac symptoms, despite being admitted to the hospital 72 h earlier. Autopsy revealed no significant macroscopic changes, and histologic examination revealed focal lymphocytic myocarditis with myocyte disruption, diffuse alveolar damage in the exudative phase, and generalized lymphocytic immune activation in other organs. Ante-mortem and post-mortem microbiological exams did not clearly prove a causative role of infectious agents. The peculiarity of this case was characterized by the contrast between the severe clinical features and the mild cardiac histological findings. This discrepancy, coupled with the suspicion of a viral causative role based on both ante-mortem and post-mortem microbiological examinations, presented significant challenges in reaching an etiological diagnosis. This case also confirms that the diagnosis of myocarditis in children cannot be made solely on the basis of histological cut-offs or microbiological results. Using abductive reasoning, various diagnostic hypotheses were formulated and evaluated to arrive at the final diagnosis of fatal myocarditis of viral or post-viral origin. Data from post-mortem examination are often the only source of information that is available to the experts, especially in cases of sudden infant death syndrome. In such cases, the forensic pathologists should accurately evaluate findings that may appear to indicate a different etiology, and, in the absence of clinical or radiological data, interpret post-mortem data in a logically correct manner. The autopsy is the first essential step to evaluate the cause of death and must be integrated with the results of ante- and post-mortem diagnostic tests in a holistic approach, which is crucial to allow forensic pathologists to provide an appropriate and relevant opinion.

## Introduction

Myocarditis is an inflammatory disease of the myocardium. The first clinical manifestations are usually palpitation or syncope, chest pain, acute heart failure with dilated cardiomyopathy, or sudden death due to arrhythmias. The occurrence of fatal myocarditis in infancy ranges from 1.59/100.000 to 1 year, 0.24/100.000 in 1–4 years, and 0.12/100.000 between 5 and 14 [1].

The most common cause of infantile myocarditis is viral; parvovirus B19 and human herpes virus 6 (HHV-6) are the most frequent pathogens in Western countries [2, 3]. Myocarditis may also be caused by bacteria, fungi, parasites, allergic drug reactions, exposure to environmental chemicals, autoimmune disorders, and hereditary diseases [4]. Finding the cause of myocarditis can be difficult, and in some cases, it is defined as idiopathic [5]. In fact, despite the detection of an infectious agent by real-time polymerase chain reaction (RT-PCR), it may be an innocent bystander with no causal relationship to myocarditis [6].

Suspicion of myocarditis is based on clinical criteria supported by troponin-T, electrocardiogram (ECG), echocardiography, cardiovascular magnetic resonance, and endomyocardial biopsy (EMB) [5, 7]. The latter is rarely used because of the high risk of cardiac perforation and valvular damage [8].

Autoptic diagnosis of myocarditis is extremely challenging, as the results obtained only from post-mortem analyses, namely gross examination, histology, or microbiology, may be ambiguous and are still controversial [8, 9]. When available, integration with ante-mortem data (blood chemistry, clinical presentation, and radiology) is mandatory to define the cause of death.

This is what happened in the present case: the death of a 23-month-old child clinically diagnosed with myocarditis who deteriorated rapidly and died 72 h after admission. The clinical presentation, histological features, and results of ante- and post-mortem microbiological tests are discussed, together with the pitfalls of post-mortem examination.

## Case report

### Clinical history

A 23-month-old child was hospitalized for a high fever (39 °C), pharyngitis, aphthous stomatitis, and vomiting. The test for group A beta-hemolytic streptococcus (GABHS) was negative. He had previously had four recurrent episodes of fever, possibly viral in origin, lasting less than 24 h each over a period of 2 months.

After 48 h, the child presented asthenia and polypnea. Blood exams revealed high levels of troponin-T (225 ng/L) and C- reactive protein (CRP) (98.5 mg/L). A multiparametric molecular test (FilmArray BioFire) on bronchoalveolar lavage was positive for enterovirus/rhinovirus and negative for adenovirus, SARS-COV-2, influenza A, influenza B, parainfluenza, respiratory syncytial virus, Mers-Cov, metapneumovirus, bordetella pertussis, bordetella parapertussis, chlamydophila, and mycoplasma pneumoniae.

The patient was IgG positive and IgM negative for cytomegalovirus, HHV-6, and Epstein-Barr virus (EBV), while no antibodies (IgG and IgM) for enterovirus were detected. Low level of HHV6 DNA (476 copies/mL) was detected in the blood by RT-PCR. Echocardiography showed severe hypokinesia and moderate left ventricular wall dilatation, raising the suspicion of myocarditis. Pulmonary echography revealed edema. Dopamine and dobutamine were administered, and the patient was transferred to the intensive care unit, but the clinical condition worsened with tachypnea, pulmonary crackles, pinkish and foamy sputum, a low pulse rate (< 60 bpm), sensory clouding, and skin mottling.

Cardiac arrest occurred 65 h after admission; cardiopulmonary resuscitation with adrenaline infusion restored circulation after 2 min. Severe bradycardia and electromechanical dissociation occurred 72 h after admission. The child died after 50 min of cardiopulmonary resuscitation. The public prosecutor asked the forensic pathologist to determine the cause of death.

### Autopsy

The autopsy was performed according to the guidelines for the autopsy investigation of sudden cardiac death developed by the Association for European Cardiovascular Pathology [10].

No traumatic injuries or malformations were identified. The heart was of normal shape and weight for age (65 g). The epicardium was smooth, and epicardial fat was normal. At sequential segmental analysis, there was the usual arrangement of heart chambers and great vessels [11].

Transverse sections across the ventricles showed mottling of the inferolateral wall of the left ventricle and the septum, while the trabecular myocardium itself appeared pale. The thickness of the parietal myocardium of the right ventricle was 0.4 cm in the upper third, 0.3 cm in the middle third, and 0.2 cm in the lower third. The parietal myocardial thickness of the left ventricle was 0.8 cm in the upper third and 0.7 cm in the middle third and in the lower third. The interventricular septum was thickened, measuring 0.7 cm in the upper and middle thirds and 0.6 cm in the lower third. The right lung weighed 171 g and the left 160 g. The lungs were congested. There were no other significant pathological findings.

### Histology

**Fig. 1:**
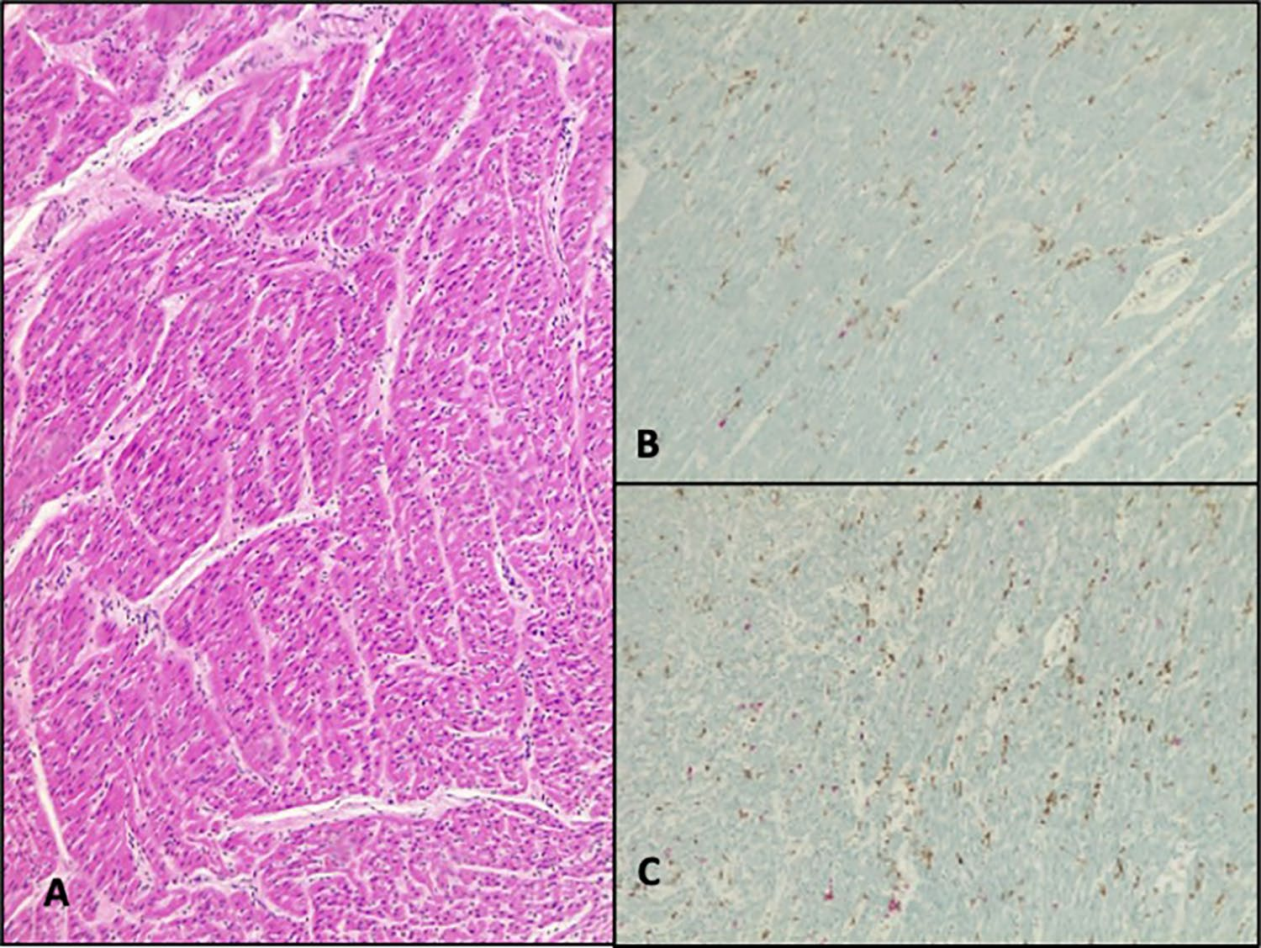
Lymphocytic myocarditis: scattered lymphocytes were present in all sections (**A**, HE staining, 10 HPF), also evidenced by immunohistochemistry for CD3 (T-cells, red) and CD68 (macrophages, brown). Sections were taken from the left ventricle (**B**, 10HPF) and septum (**C**, 10 HPF)

**Fig. 2:**
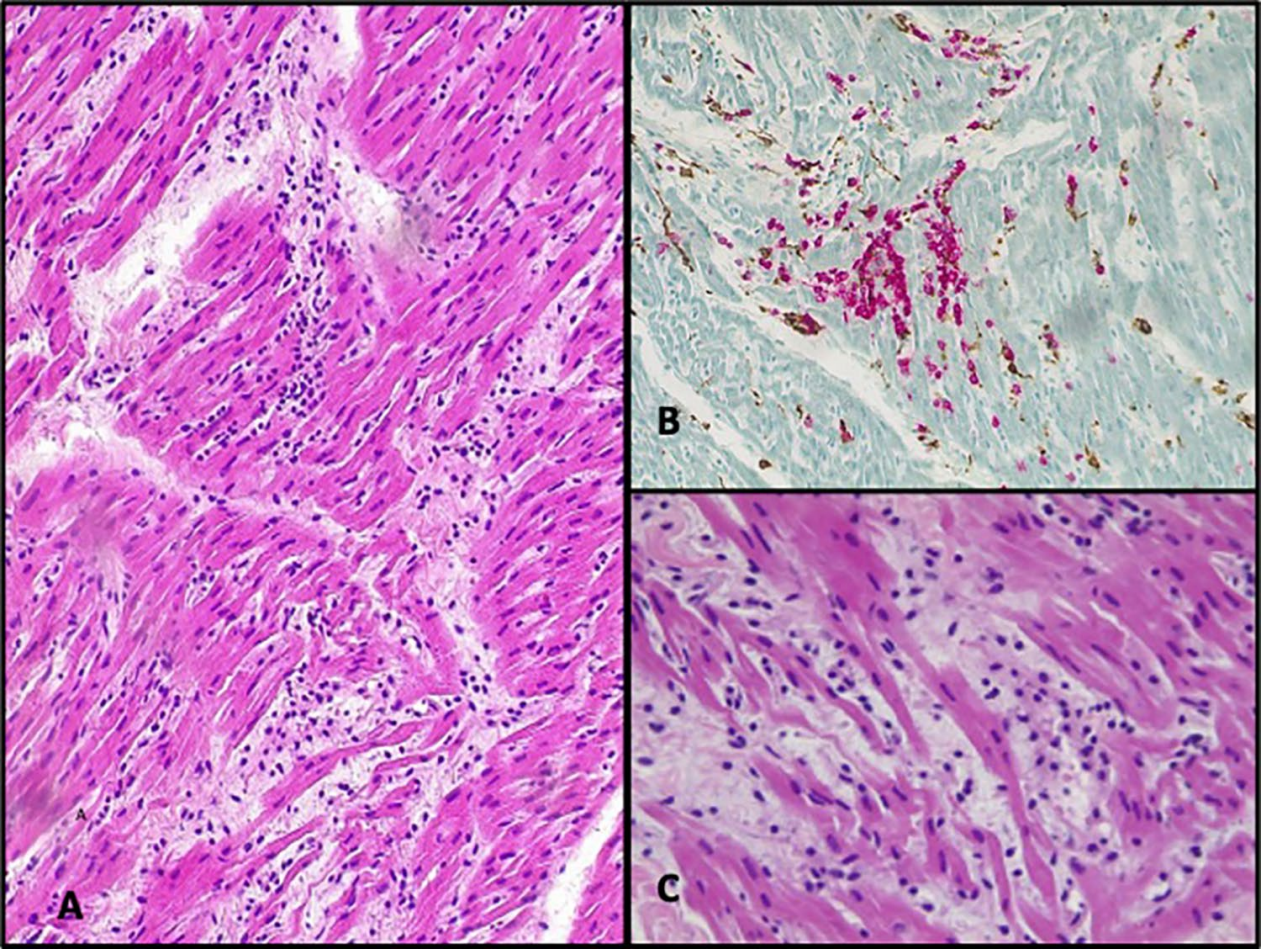
Lymphocytic myocarditis: only one focus presented more than 14 T cells/mm2 (**A**, HE staining, 20 HPF), evidenced by immunohistochemistry for CD3 (T-cells, red) and CD68 (macrophages, brown) (**B**, 20 HPF). Myocyte damage was associated with the inflammatory infiltrate (**C**, HE staining, 40 HPF)

**Fig. 3:**
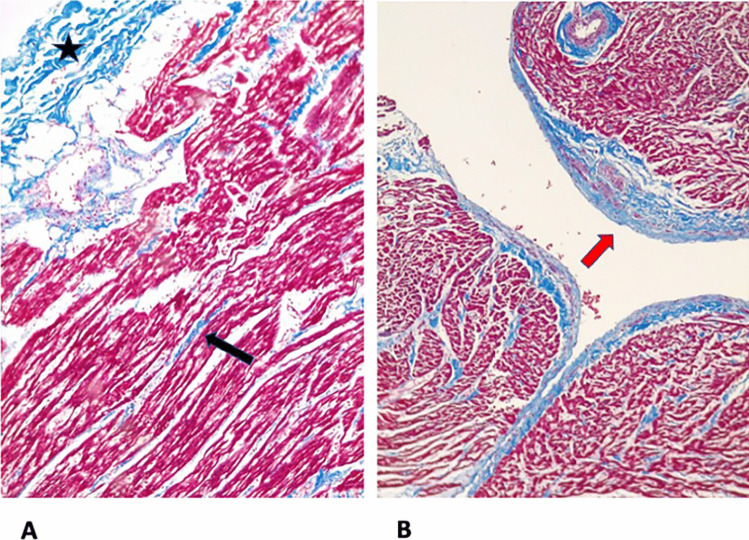
Minimal fibrosis was observed in the epicardium (star), in the interstitium (arrow) (**A**, trichrome stain, 10 HPF), and in the endocardium (**B**, trichrome stain 10 HPF)

The heart was thoroughly sampled with 7 sections from the free wall of the left ventricle, 2 from the free wall of the right ventricle, 2 from the septum, and one full section at the apex. A focal interstitial inflammatory infiltrate, composed of lymphocytes and histiocytes, was present in all sections, with no evidence of zonal distribution. There was focal and minimal involvement of the endocardium and epicardium. Double immunohistochemistry for TCD3 (Dako, polyclonal) and CD68 (Thermofisher, monoclonal) confirmed lymphocytic myocarditis with less than 7 T cells/mm^2^ in most of the fields (Fig. 1). Only one focus showed more than 14 T cells/mm^2^ associated with myocyte damage, detected in the right ventricle in the superior part (Fig. 2). This was classified as grading 4/5 and staging 2/5 according to the classification proposed by Basso et al. [6]. There was no evidence of acute myofiber necrosis. Fibrosis was minimal, located in the epicardium, endocardium, and interstitium (Fig. 3).

The lungs showed diffuse alveolar damage in the exudative stage with abundant oedema, intra-alveolar neutrophils, and hyaline membranes. Immunohistochemistry for C4d (cell marque, polyclonal) was diffusely positive.

**Fig. 4:**
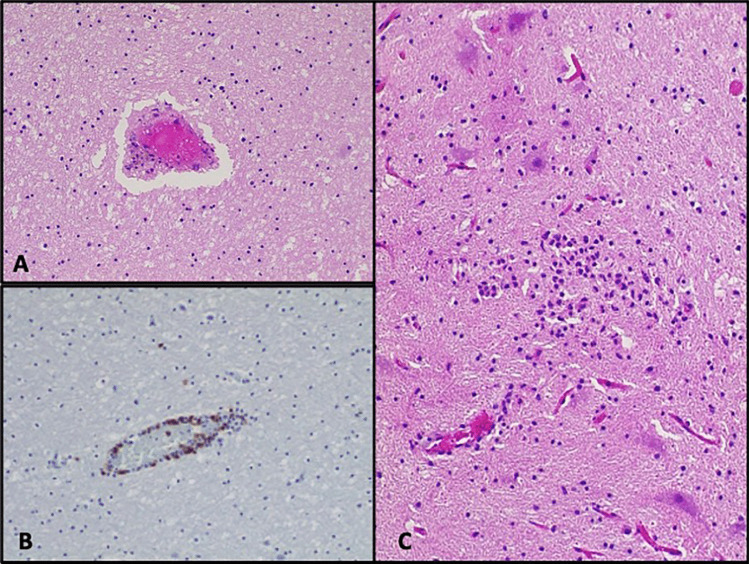
Cerebral perivascular lymphocytic infiltration (**A**, HE 20 HPF), evidenced by immunohistochemistry for CD3 (**B**, T-cells, brown, 20 HPF). A microglial nodule was also observed in the white matter (**C**, HE staining, 20 HPF)

The other tissues showed a widespread lymphocytic infiltrate with prominent lymphoid follicles in the spleen, tonsillae, and mediastinal lymph nodes. There was also increased lymphocytic infiltration of the gastro-enteric tract. In the brain, there were diffuse perivascular lymphocytes and focal microglial nodules (Fig. 4). No lymphocytic infiltrates were found in the solid organs.

### Microbiological findings

Post-mortem microbiological exams were carried out on frozen myocardial tissue. RT-PCR was negative for CMV DNA, HSV1 e HSV2 DNA, adenovirus DNA, SARS-COV-2 RNA, parvovirus B19 DNA, enterovirus RNA, and EBV DNA. A weak positive result for HHV6 DNA < 10 copies/µg di DNA was found.

RT-PCR on formalin-fixed paraffin-embedded (PPFE) lung was negative for the same viruses. Positive results were detected for HHV6 and EBV DNA (both under the lower limit of quantification < 10 copies/µg).

## Discussion

We described a case of sudden death in a previously healthy child, who died 72 h after hospital admission. The peculiarity of this case was characterized by the contrast between the severe clinical features and the mild cardiac histological findings. This discrepancy, coupled with the suspicion of a viral causative role based on both ante-mortem and post-mortem microbiological examinations, presented significant challenges in reaching an etiological diagnosis.

On post-mortem examination, abductive reasoning [12] suggested two possible causes of death: myocarditis causing the diffuse alveolar damage or an undetermined lung disease. Even though the lung tissue was severely damaged on microscopic examination, suggesting diffuse alveolar damage, there were relevant ante-mortem data that strongly supported a cardiac etiology as the precipitating event. In fact, the child presented acute cardiological signs and symptoms (asthenia, cyanosis of lower limbs, tachycardia, pulmonary edema), with a rapid deterioration leading to heart failure in less than 24 h, and then developing an acute respiratory distress syndrome (ARDS), which was the final event. The cardiac etiology was also supported by the high levels of troponin-T (225 ng/L) and by the severe hypokinesia with moderate dilatation of the left ventricular wall detected on echocardiography 48 h after the onset of the symptoms. However, some results of the post-mortem examinations, namely the mild histological features and the positive virological results, were not consistent with the hypothesis of viral myocarditis and should be discussed carefully.

Regarding the histologic findings, some cut-offs have been proposed for the diagnosis of myocarditis. Basso et al. considered a value > 14 leukocytes/mm2, with the presence of T-lymphocytes > 7 cells/mm2 a realistic cut-off to reach a diagnosis of myocarditis [6]. In our case, the myocardium showed multifocal inflammatory infiltrate, but in most of the fields there were less than 7 T-cells/mm2, with only one focus with more than 14 T-cells/mm2. The inflammatory infiltrate in the present case in most of the fields was also lower than the cut-off proposed by Dettmeyer et al. [13] for the diagnosis of infantile myocarditis (> 15 leukocytes or > 10 T-lymphocytes). However, Grasmeyer et al. [9] demonstrated the low applicability of these cut-offs to postmortem samples, at least in infants, suggesting that the diagnosis of myocarditis from autopsy tissue still requires the application of the well-established Dallas Criteria [14], which define the histological evidence of an “inflammatory infiltrate of the myocardium with necrosis and/or degeneration of adjacent myocytes.” More recently a score was proposed, containing, beside histology, also macroscopic findings and anamnestic information, such as cardiac symptoms [15]. Accordingly, in this case, the inflammatory infiltrate of the myocardium, if integrated with clinical, radiological, and laboratory findings, is strongly suggestive for a myocarditis.

As for the interpretation of microbiological results, a multiparametric molecular test performed ante-mortem on BAL detected enterovirus/rhinovirus RNA. It was not possible to establish if enterovirus/rhinovirus infection was prior to hospitalization or secondary to clinical practices, as they can occur after intubation procedures [16]. Moreover, enterovirus/rhinovirus RNA was probably not of clinical relevance, as serology was negative. The RT-PCR performed on frozen myocardial tissue and on formalin-fixed paraffin-embedded (FFPE) lung resulted negative for enterovirus suggesting that positive result obtained on BAL was related to rhinovirus. It is known that HRV infection is usually mild and self-limited. However, in some pediatric cases, it is responsible for severe symptoms, such as wheezing, exacerbation of asthma, bronchiolitis, severe pneumonia, and cardiopulmonary disease [17]. The low positive results obtained for EBV and HHV-6 in tissues are related to cells harboring the viruses in a latent form [18].

To better understand the etiology of myocarditis in the present case, several aspects need to be considered. The child had prodromal symptoms (several episodes of high fever, pharyngitis, and stomatitis), which are typical of a viral infection. At histology, the inflammatory infiltrate was mainly composed of T-CD3 lymphocytes and macrophages, which are typically associated with idiopathic (post-viral) or viral infections [19]. This hypothesis was also supported by several histological signs of diffuse activation of the lymphocytic immune system, which were most likely due to a previous infectious event or viral etiology, namely diffuse lymphocytic infiltration of the gastro-enteric tract, follicular splenic and tonsillar hyperplasia, cerebral perivascular lymphocytic infiltrate, and follicular hyperplasia of the mediastinal lymph nodes. Therefore, histological evidence was suggestive of an infective agent, probably viral, that triggered the outbreak of myocarditis, favoring or leading to the pulmonary damage [20]. There are also some explanations for the negative viral exams: the presence of the microorganism may not be detected postmortem because of degradation of viral DNA/RNA and the low sensitivity of RT-PCR on FFPE tissues [4, 21]. Also, the quantity of the tissue analyzed may be too small [6], or the identifying panel is too limited [4], or the microorganisms might have been cleared by the immune response (post-viral myocarditis). In fact, because of the cytolytic effect of the virus, intracellular or surface antigens may be released and trigger an autoimmune response, also activated by a cross-priming pathway when dying cardiac cells are engulfed by antigen-presenting cells (APCs) [22]. Finally, in our case, there was no evidence to suggest other probable causes besides viral or post-viral.

The integrated multidisciplinary approach, based on clinical history, imaging and blood chemistry data, autopsy findings, and histological examination, allowed the cause of death to be identified as acute respiratory distress syndrome with diffuse alveolar damage, probably caused by lymphocytic myocarditis of viral (rhinovirus or not investigated viruses) or post-viral origin.

Given the intricate nature of scenarios related to viruses and the questionable histological cut-offs for infant death in myocarditis, this case demonstrates the need for an integrated and multidisciplinary approach, in order to understand the etiopathogenesis of sudden cardiac death, which can be extremely challenging, especially in the forensic scenario [23-25].

A significant issue in forensic is that data from post-mortem examinations are often the only source of information that is available to the experts, especially in cases of sudden infant death syndrome. In these cases, the forensic pathologist should carefully evaluate findings that may suggest an etiology other than myocarditis and, in the absence of clinical, radiological, or laboratory data, interpret post-mortem data in a logical manner. The aim of our report is to highlight that in the case of fatal pediatric myocarditis, post-mortem analysis may give uncertain results that should be carefully evaluated, especially in the absence of ante-mortem data, as is often the case in forensic scenarios (e.g., SIDS). The post-mortem diagnosis of myocarditis is based on a thorough autopsy examination and exclusion of other causes, as well as the demonstration of myocyte damage associated with a lymphocytic inflammatory infiltrate.

In conclusion, our report highlights that in a complex scenario, a comprehensive forensic investigation is mandatory to understand the cause and the cause and the manner of death. The autopsy is the first essential step in assessing the cause of death and must be integrated with the results of ante- and post-mortem diagnostic tests in a holistic approach, which is crucial for forensic pathologists to provide an appropriate and relevant opinion that will allow the court to reach the correct verdict.

## Key points

1. Diagnosis of myocarditis in children cannot be made solely on the basis of histological or microbiological results
2. Some cases of myocarditis are clinically severe despite mild cardiac histological findings
3. The autopsy must be integrated with the results of ante- and post-mortem diagnostic tests
4. Abductive reasoning is necessary to interpret post-mortem data in a logically correct manner

## Data Availability

The data presented in this study are available on request from the corresponding author.
